# Pulmonary embolism causing atrial fibrillation with slow ventricular response: a case report

**DOI:** 10.1186/s12872-019-1081-8

**Published:** 2019-04-29

**Authors:** Mohammad A. Abdulsalam, Mohammed N. Elganainy, Ahmad J. Abdulsalam

**Affiliations:** 1 Department of Internal Medicine, Mubarak Al Kabeer Hospital, Jabriya, Kuwait; 2 Department of Cardiology, National Heart Institute, Cairo, Egypt; 3 Resident in the Kuwaiti Board of Physical Medicine and Rehabilitation, Physical Medicine and Rehabilitation Hospital Kuwait, Street 104, Andalous, Kuwait

**Keywords:** Atrial fibrillation, Arrhythmia, Case report, Pacemaker, Pulmonary embolism, Slow

## Abstract

**Background:**

Pulmonary embolism (PE) is a fatal condition, with a subsequent variety of complications. Although rare, the ensuing presentation of atrial fibrillation (AF) secondary to PE is evident in the literature. However, there has been no report of AF with slow ventricular response requiring a pacemaker as a complication of PE.

**Case presentation:**

A 78-year-old obese female presented to the emergency room with new onset dyspnea. Computed tomography pulmonary angiogram revealed bilateral PE. Twenty-four hours later, the patient developed new onset AF with slow ventricular response. Therefore, a single chamber pacemaker was implanted.

**Conclusion:**

PE causing AF with slow ventricular response has not been reported or explained in the literature. The mechanism of this complication is yet to be understood and will require further investigation to explain this newly presented relationship.

## Background

Pulmonary embolism (PE) is a fatal condition, which can cause a variety of complications. One of these rare complications are arrythmias such as atrial fibrillation (AF) [1]. Although rare, the ensuing presentation of AF secondary to PE is evident in the literature, however there has been no report of PE causing AF with slow ventricular response requiring a pacemaker [1–3]. We present a case of AF with slow ventricular response caused by a bilateral PE, with a conceivable pathophysiological hypothesis.

## Case presentation

**Fig. 1:**
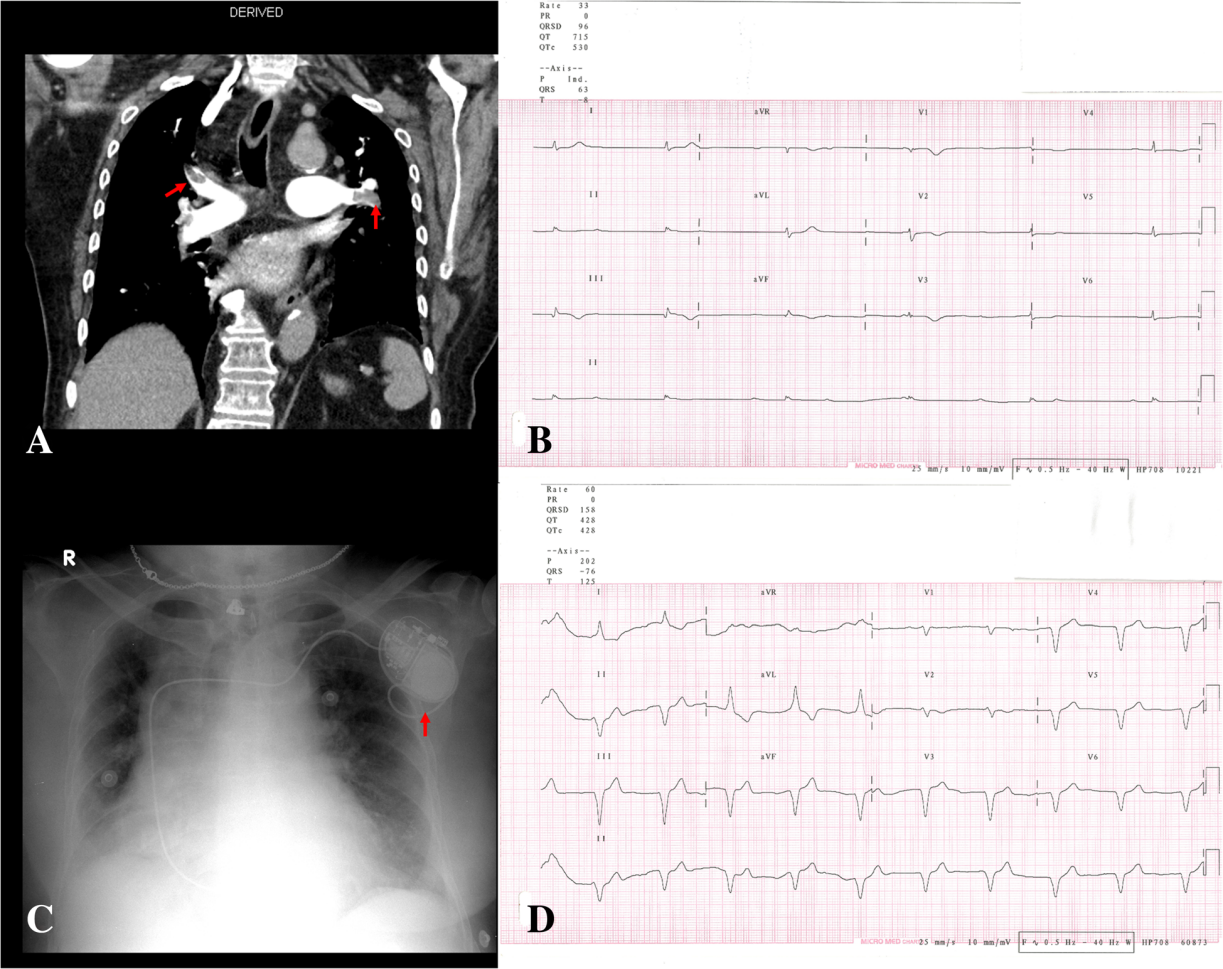
**a** Computed tomography pulmonary angiography revealing filling defects (red arrow) in the distal left and right main pulmonary arteries **b** Twelve lead electrocardiograph showing atrial fibrillation with ventricular response of 33 beats per minute. **c** Chest x-ray after implantation of a single chamber pacemaker (red arrow) in the left pectoral pocket. **d** Twelve lead electrocardiograph post pacemaker implantation

A 78-year-old obese female presented to the emergency room with new onset dyspnea of one day duration, which worsened in the past couple of hours. Her medical history included hypertension and a hemorrhagic stroke two years prior which left her bedbound. She denied any familial history of PE, leg pain, or palpitation. At admission, blood pressure, pulse rate and peripheral oxygen saturation were 116/78 mmHg, 135 beats/min and 88%, respectively. On physical examination, she had tachypnea (30 breaths/minute) and electrocardiography revealed sinus tachycardia. Arterial blood gas analysis on room air yielded pH 7.44, PCO2 33.9 mmHg, and PO2 72.9 mmHg. Routine blood tests demonstrated a normal cardiac troponin I levels and no evidence of electrolyte imbalances, while chest X-ray revealed no signs of heart failure. Nevertheless, D-dimer was highly elevated (> 4000 ng/dL) increasing the suspicion of PE. Computed tomography pulmonary angiogram was sought revealing bilateral PE (Fig. 1a). Lower limb Doppler was negative for deep vein thrombosis.

Twenty-four hours after diagnosing bilateral PE and stabilizing the patient with anticoagulation and hemodynamic support, the patient developed new onset palpitation, dizziness, and fatigue. Cardiac enzymes were repeated showing a mild elevation. Electrocardiography reveled new onset AF with slow ventricular response of 33 beats/min (Fig. 1b). She was not on any negative chronotropic drugs and no electrolyte imbalance was detected. Echocardiography revealed normal left ventricular systolic function and dimensions, left ventricular regional wall motion, and both left and right atrium dimensions. However, it highlighted dilated right ventricular dimensions with a basal diameter of 50 mm and evidence of McConnell’s sign (right ventricular free wall hypokinesia) with paradoxical septal wall motion. In addition, it revealed impaired right ventricular systolic function with tricuspid annular plane systolic excursion of around 1.5 cm, flattening of intraventricular septum, and moderate tricuspid regurgitation with pulmonary artery systolic pressure around 50 mmHg. As the patient was developing hemodynamic instability, 48 h later, single chamber pacemaker was implanted in the left pre-pectoral pocket and the basal heart rate was set up to 60 beats per minute (Fig. 1c and d). After a 2-month follow-up, the patient showed normal vital signs and her electrocardiogram showed a paced rhythm with a heart rate of 60 beats per minute. She developed no further complications or clinical morbidities in spite her poor prognosis.

## Discussion and conclusion

The association of causation between PE and AF with slow ventricular response remains to be vague in the literature in which the exact pathophysiology and causal mechanism is yet to be fully understood [2, 3]. On a pathophysiological level that might demonstrate AF, Flegel concluded that a PE may trigger AF by causing acute right ventricular dilatation with strain, something that was evident in our case [4]. Another case by Marti et al. hypothesized about a similar pathology stating that a PE could trigger an adrenergic surge stimulating the Bezold-Jarish reflex, a vagal response and decreased sympathetic tone, leading to abnormal heart electrical conduction which in part may cause slow conduction [5]. Another plausible hypothesis for the slow conduction is that the massive PE may have caused transient myocardial ischemia and dysfunction to the atrioventricular node. The consequence of this massive PE was demonstrated in the echocardiography as regional pattern of right ventricular dysfunction, with akinesia of the mid free wall and hyper contractility of the apical wall, which is also known as McConnell’s sign [6]. We believe the conclusion of Flegel along with one of the latter two hypothesis may be an explanation of the AF with slow ventricular response caused by PE [4, 5].

To our knowledge, PE causing AF with slow ventricular response has not been reported or explained in the literature. Hence, the mechanism of this complication is yet to be understood and will require further investigation to explain this newly presented relationship.

## Abbreviations

AF: Atrial fibrillation
PE: Pulmonary embolism
